# Layered double hydroxide-oxidized carbon nanotube hybrids as highly efficient flame retardant nanofillers for polypropylene

**DOI:** 10.1038/srep35502

**Published:** 2016-10-18

**Authors:** Yanshan Gao, Yu Zhang, Gareth R. Williams, Dermot O’Hare, Qiang Wang

**Affiliations:** 1 College of Environmental Science and Engineering, Beijing Forestry University, 35 Qinghua East Road, Haidian District, Beijing 100083, P. R. China; 2 UCL School of Pharmacy, University College London, 29-39 Brunswick Square, London WC1N 1AX, United Kingdom; 3 Chemistry Research Laboratory, Department of Chemistry, University of Oxford, Mansfield Road, Oxford OX1 3TA, United Kingdom

## Abstract

Aqueous miscible organic layered double hydroxides (AMO-LDHs) can act as organophilic inorganic flame retardant nanofillers for unmodified non-polar polymers. In this contribution, AMO [Mg_3_Al(OH)_8_](CO_3_)_0.5_·*y*H_2_O LDH–oxidized carbon nanotube (AMO-LDH–OCNT) hybrids are shown to perform better than the equivalent pure AMO-LDH. A synergistic effect between the AMO-LDH and OCNT was observed; this endows the hybrid material with enhanced flame retardancy, thermal stability, and mechanical properties. The thermal stability of polypropylene (PP) was significantly enhanced by adding AMO-LDH–OCNT hybrids. For PP mixed with AMO-LDH–OCNT hybrids to produce a composite with 10 wt% LDH and 2 wt% OCNT, the 50% weight loss temperature was increased by 43 °C. Further, a system with 10 wt% of AMO-LDH and 1 wt% OCNT showed a peak heat release rate (PHRR) reduction of 40%, greater than the PHRR reduction with PP/20 wt% AMO-LDH (31%). The degree of dispersion (mixability) between AMO-LDH and OCNT has a significant effect on the flame retardant performance of the hybrids. In addition, the incorporation of AMO-LDH–OCNT hybrids led to better mechanical properties, such as higher tensile strength (27.5 MPa) and elongation at break (17.9%), than those composites containing only AMO-LDH (25.6 MPa and 7.5%, respectively).

Polypropylene (PP) is one of the most widely used commodity thermoplastics, and has excellent physical properties such as high stiffness and tensile strength^1^,^2^. However, it has a low thermal stability and is a flammable material. In order to improve its flame retardant properties, various types of inorganic nanofillers have been introduced, such as Al(OH)_3_^3^, Mg(OH)_2_^4^, carbon nanotubes (CNTs)^5^,^6^, layered double hydroxides (LDHs)^7^,^8^,^9^, and zinc borate^10^, among others. From the studies conducted to date, it is clear that the main drawbacks of inorganic flame retardant nanofillers are the high loading required, their low efficiency, and their inherent poor compatibility with the polymer matrix. The mechanical properties of PP may also be compromised by the presence of large amounts of inorganic fillers. Thus, current research seeks to decrease the loading required while maintaining appropriate flame retardant performance.

In recent years, LDHs have attracted increasing attention as a new generation of environmentally friendly and highly efficient flame retardants for polymers, due to their tunable chemical compositions and layered structures^11^,^12^,^13^,^14^,^15^. In our previous studies, we have demonstrated that both the thermal and flame retardant properties of PP, high density polypropylene (HDPE), and ethylene–vinyl acetate copolymer (EVA) can be greatly improved by the addition of a new generation of hydrophobic and hydrocarbon–dispersible LDHs called aqueous miscible organic LDHs (AMO–LDH)^16^,^17^,^18^. For instance, by adding 40 wt% of AMO-Zn_2_Al–SO_4_ LDH into HDPE, the peak heat release rate (PHRR) could be decreased from 1521 to 706 Wg^–1^ (a 54% reduction in PHRR)^16^. Jiao *et al*.^19^ reported that the flame retardant performance of EVA resin could be improved to some extent by adding ca. 50 wt% of Mg/Al–CO_3_ or Mg/Al/Fe–CO_3_ LDHs. Further, Qu *et al*.^20^ proved that an EVA sample with 55 wt% of MgAl–PO_4_ LDH can reach the UL–94 V–1 rating. Although LDHs have thus been proven to be efficient as flame retardant nanofillers for polymers, the required loadings are still too high for commercial application (normally up to 40–60 wt%). In order to reduce the LDH loading required while also maintaining or improving the flame retardant efficiency, one option is to add a combination of fillers such as CNTs together with LDH.

The use of CNTs as flame retardant nanofillers for polymers was first reported by Ajayan *et al*. in 1994^21^. Subsequently, several related studies have been conducted^22^,^23^,^24^. For example, Kashiwagi *et al*.^25^,^26^ have demonstrated that the PHRR of PP could be reduced by 27 or 32% by adding 1 or 2 vol.% of CNTs, respectively. It was also found that a networked layer of CNTs forms during burning. This covers the entire surface of the sample without forming any significant cracks. Other studies have demonstrated that the dense network of nonflammable CNTs acts as a physical barrier to the diffusion of oxygen and also slows the escape of combustion products formed during decomposition, which can shield the polymer resin from external radiation and heat feedback from the flame^26^,^27^,^28^. However, although CNTs have shown good potential in flame retardancy their dispersion within the polymer matrix is normally poor, due to their hydrophilic nature.

Considering the fact that LDHs and CNTs possess different flame retardant mechanisms, the potential for synergistic effects between them when they coexist in polymers is of great interest. Thus, in this contribution, AMO-LDH and oxidized CNT (OCNT) hybrids were prepared using a hydrothermal method. The AMO-LDH–OCNT hybrids were then introduced into PP using a solvent mixing method to produce a range of composites (see Table 1). All the AMO-LDHs, AMO-LDH–OCNT hybrids, and PP based nanocomposites were thoroughly characterized. Finally, the thermal and flame retardant performances as well as the mechanical properties of the PP/AMO-LDH–OCNT nanocomposites were compared with those of both PP/AMO-LDH and PP/OCNT nanocomposites.

## Results and Discussion

Due to their large length−to−diameter ratio and high surface energy, CNTs twist around themselves, which can hamper their applications. Therefore, CNTs were first modified (oxidized) with concentrated nitric acid to be OCNT before the preparation of AMO-LDH–OCNT hybrids and polymer/AMO-LDH–OCNT nanocomposites. Active oxygen atoms may be released during the nitric acid treatment process, and surface bound –C = O groups are formed. These are then oxidized to surface –COOH groups.

**Figure 1:**
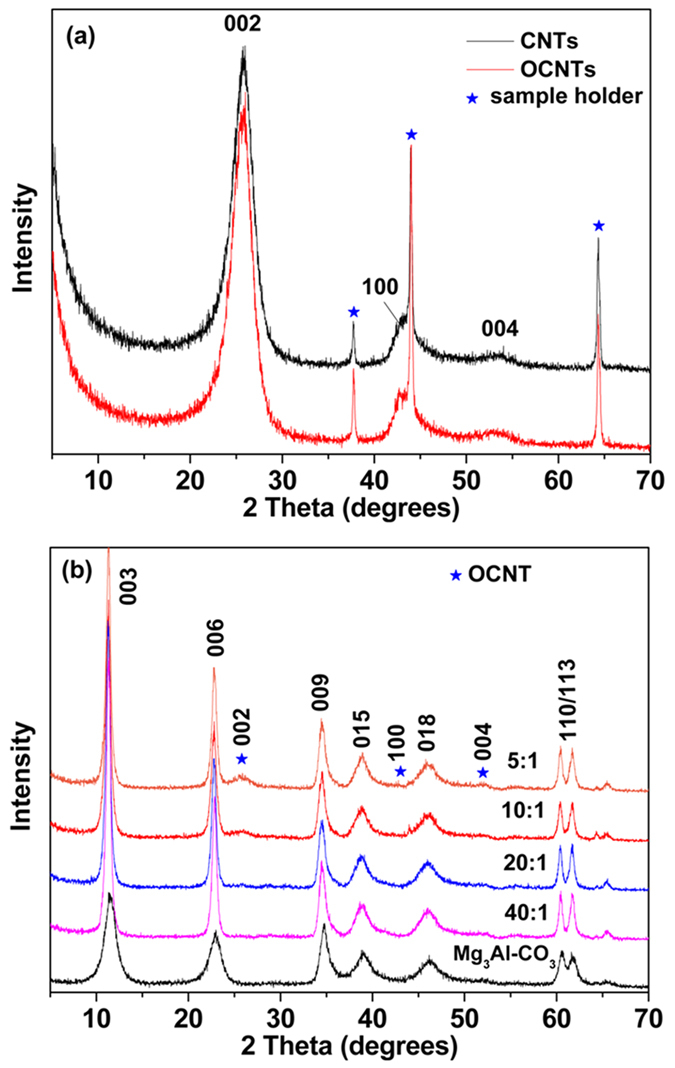
XRD patterns of (**a**) CNT and OCNT, and (**b**) AMO-LDH and AMO-LDH‒OCNT hybrids with different AMO-LDH/OCNT ratios.

The CNTs and OCNTs were characterized using X–ray diffraction (XRD) analysis, as shown in Fig. 1(a). Three characteristic reflections at 2θ = 26°, 43°, and 54° corresponding to the Bragg reflections (002), (100), and (004) were observed, which is consistent with literature reports^29^,^30^. After oxidation, the characteristic reflections of the OCNTs are identical to those reported for CNT samples, suggesting the long–range structure of the CNTs has not changed as a result of oxidation.

The XRD patterns of AMO-LDH and various AMO-LDH–OCNT hybrids are given in Fig. 1(b). Both pristine AMO-LDH and the hybrids exhibited the typical reflections of hydrotalcite–like materials, with pronounced basal (00*l*) harmonics, indicating a well–crystallized structure for the AMO-LDH nanoparticles. After hybridization, the (002), (100), and (004) Bragg reflections from OCNTs (★) gradually increased in intensity with increasing OCNT loading. These results indicate that AMO-LDH-OCNT hybrids have been successfully formed. The unit cell parameters derived from XRD data are summarized in Table 2. The unit cell parameter, *a* corresponds to the metal–metal distance within the layer and the *c* parameter is related to the distance between layers^31^. These parameters can be calculated through the relationships *a* = 2d_110_ and *c* = 3d_003_^32^. Almost constant values for both *a* and *c* were obtained, demonstrating that the nature of the AMO-LDH, which is reflected in both the metal–metal distance in the brucite–like layers and the interlayer distance, was not disturbed by the introduction of OCNTs^33^.

**Figure 2:**
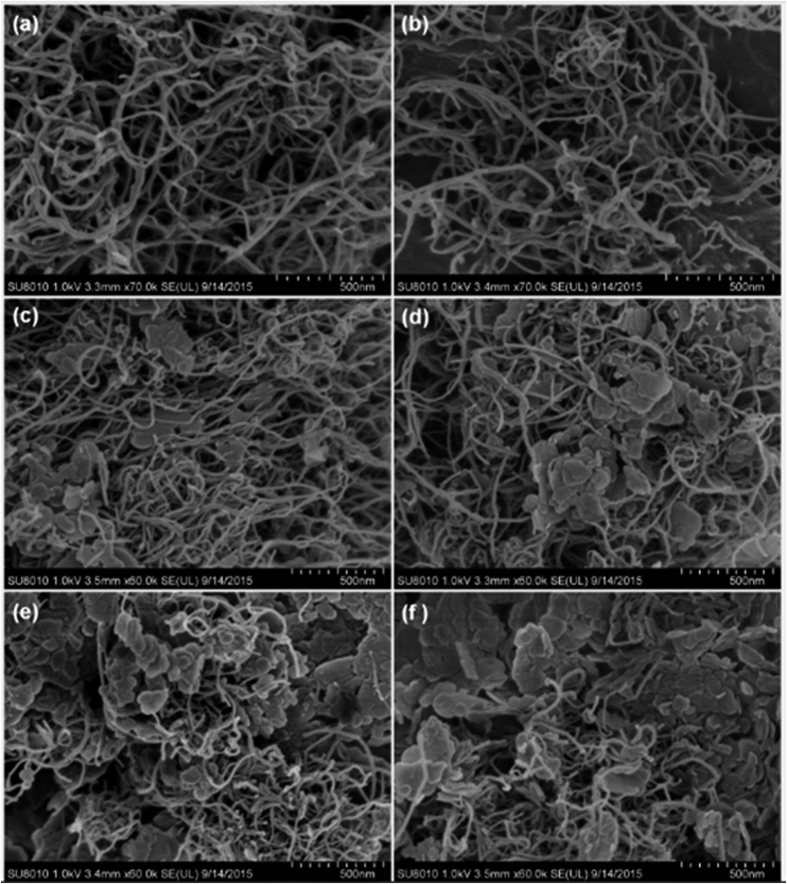
SEM images of (**a**) CNTs, (**b**) oxidized CNTs, and AMO-LDH‒OCNT hybrids with an AMO-LDH/OCNT ratio of (**c**) 5:1, (**d**) 10:1, (**e**) 20:1, and (**f**) 40:1.

Figure 2(a‒b) give scanning electron microscope (SEM) images of CNTs and OCNTs. Both pristine CNTs and OCNTs possess tubular morphologies, with an average diameter of ~30–40 nm. The pristine CNTs have smooth surfaces and long lengths. The OCNTs are more commonly broken after acidification, with more tube ends being observed. The ends of the nanotubes are believed to improve the compatibility between the OCNTs and polymer matrix^34^. Figure 2(c‒f) depict images of AMO-LDH‒OCNT hybrids with various AMO-LDH to OCNT ratios. It can be seen that AMO-LDH platelets with an average lateral size of 200‒300 nm are randomly attached to the surface of OCNTs. More platelets are observed with an increase in the AMO-LDH/OCNT ratio.

**Figure 3:**
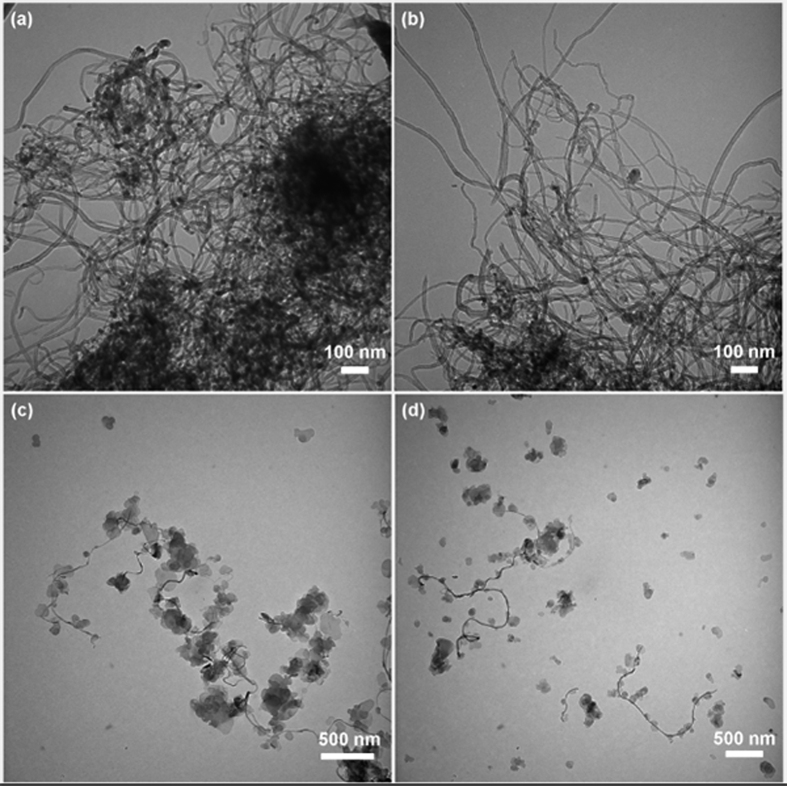
TEM images of (**a**) CNTs, (**b**) oxidized CNTs, and AMO-LDH‒OCNT hybrids with an AMO-LDH/OCNT ratio of (**c**) 10:1, and (**d**) 40:1.

AMO-LDH, CNTs, OCNTs, and AMO-LDH‒OCNT hybrids were also characterized using transmission electron microscopy (TEM). Figure 3(a‒b) include images of CNTs and OCNTs, showing both to have diameters of about 30‒40 nm. Most of the pristine CNTs are twisted around themselves forming bundles, and their tops are closed. After oxidation, most tops of the OCNTs are opened due to the reaction with concentrated acid, but the cannular structure is not changed. These results are consistent with the SEM analyses. Figure 3(c‒d) show the TEM images of the AMO-LDH‒OCNT hybrids with AMO-LDH/OCNT ratios of 10:1 and 40:1, respectively. The AMO-LDH plates are clearly attached to the surface of the OCNTs. When the AMO-LDH loading was low, almost all the AMO-LDH particles are associated with OCNTs (Fig. 3(c)). However, when the AMO-LDH/OCNT ratio was higher (40:1), not all the AMO-LDH nanoplates can be attached to the surface of the OCNTs, and more free particles were observed. Considering the morphology of the AMO-LDH‒OCNT hybrids, it is anticipated that the aggregation of CNTs within the PP matrix could be reduced owing to their being isolated from the bulk polymer by the hydrophobic AMO-LDH surfaces^33^.

**Figure 4:**
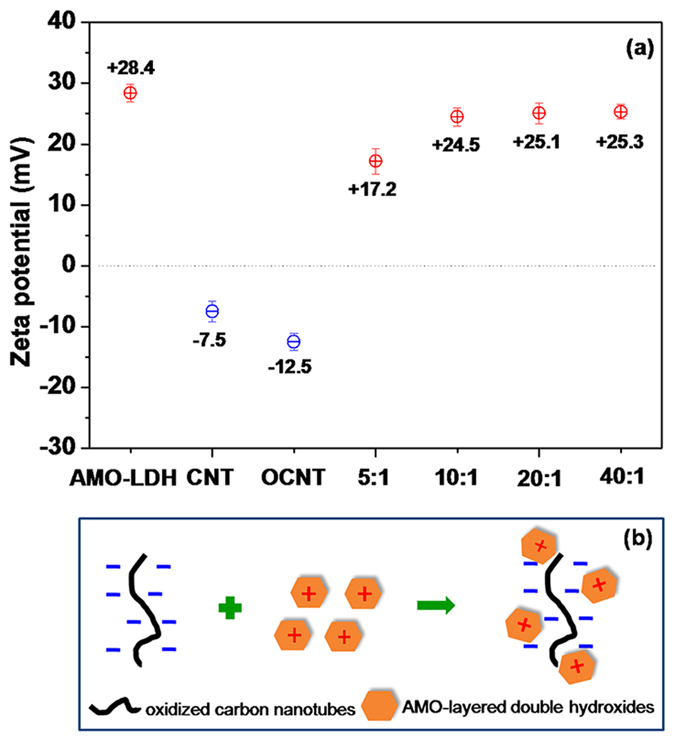
(**a**) Zeta potentials of AMO-LDH, CNT, OCNT, and the AMO-LDH‒OCNT hybrid materials in water, and (**b**) a schematic illustrating the interactions between AMO-LDH and CNTs.

The zeta potential (ζ‒potential) is an important parameter to evaluate the surface charge and colloidal stability of suspensions^35^. Values for the hybrids are detailed in Fig. 4(b). The ζ‒potential value of an AMO-LDH suspension is + 28.4 ± 1.4 mV, which results from the positive surface charge of the AMO-LDH particles^36^. In contrast, the OCNT suspension presents a ζ‒potential value of −12.5 ± 1.4 mV, more negative than a pristine CNTs suspension (−7.5 ± 1.7 mV). This observation is ascribed to the generation of ionisable surface carboxylic acid groups during chemical oxidation. Given their opposite surface charges, strong electrostatic interactions between AMO-LDH and OCNTs should facilitate the attachment of AMO-LDH nanoplates onto the OCNT surfaces. An illustration of the proposed model for the electrostatic interactions between AMO-LDH and OCNTs is shown in Fig. 4(b).

Although the ζ‒potentials of all AMO-LDH‒OCNT hybrid suspensions were positive, their values were lower than that of the neat AMO-LDH suspension. For instance, when the AMO-LDH/OCNT ratio was 5:1, the ζ‒potential was reduced to +17.2 ± 2.1 mV, due to the counterbalance of the AMO-LDH positive charge by the negatively charged OCNTs. As expected, all the ζ‒potential values became more positive with an increase in AMO-LDH loading. When the AMO-LDH/OCNT ratio was 40:1, the ζ‒potential became +25.3 ± 1.2 mV, quite close to that of neat AMO-LDH (+28.4 ± 1.4 mV).

**Figure 5:**
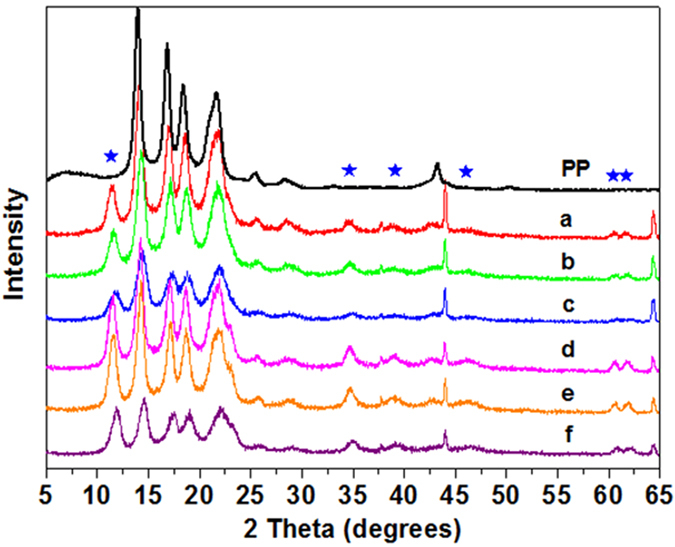
XRD patterns of pure PP and (**a**) PP/10 AMO-LDH‒0.5 OCNT, (**b**) PP/10 AMO-LDH‒1 OCNT, (**c**) PP/10 AMO-LDH‒2 OCNT, (**d**) PP/20 AMO-LDH‒0.5 OCNT, (**e**) PP/20 AMO-LDH‒1 OCNT, and (**f**) PP/20 AMO-LDH‒2 OCNT.

PP composites with different weight percentages of AMO-LDH and OCNT were prepared by mixing the polymer with the different AMO-LDH-OCNT hybrids in appropriate amounts, as detailed in the Methods section. Figure 5 illustrates the XRD patterns of pure PP and the PP/AMO-LDH‒OCNT nanocomposites. After addition of the hybrids, several new Bragg reflections are observed by XRD which can be indexed as the (003), (009), (015), (018), and (110/113) planes of AMO-LDH. This indicates that the layer structure of the materials remains intact after introduction into PP. The characteristic Bragg reflections of the OCNTs at 2θ = 26 °, 43 °, and 54 ° were not easily observed, which is because the loading of OCNTs is very low (less than 2 wt%).

**Figure 6:**
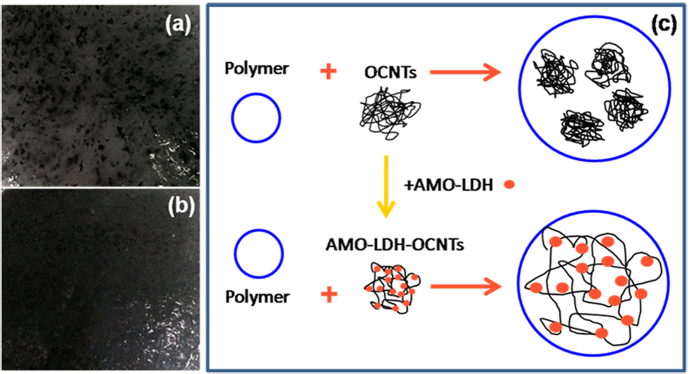
Visual appearance of (**a**) PP/OCNT and (**b**) PP/AMO-LDH‒OCNT, together with (**c**) a diagram illustrating the distribution of components in the two composites.

The extent of dispersion of OCNTs within the matrices of PP/OCNT and PP/AMO-LDH–OCNT nanocomposites are compared in Fig. 6. For PP/OCNT nanocomposites, many aggregates of OCNTs are clearly observed (Fig. 6(a)), due to the hydrophilic properties of the latter and their entwining. However, after making the AMO-LDH–OCNT hybrids, the dispersion of the OCNTs became much improved. No aggregates can be seen and a very homogeneous dispersion was obtained (Fig. 6(b)). The improved dispersion of OCNTs is thought to result from the increased hydrophobicity of the attached AMO-LDH nanoplatelets. A proposed schematic explaining the dispersion of OCNTs and AMO-LDH–OCNT hybrids within PP is illustrated in Fig. 6(c). It can be concluded that the hybridization of OCNTs with AMO-LDH nanoplatelets can prevent OCNTs from aggregating, which should enhance the flame retardant, mechanical and thermal performance.

**Figure 7:**
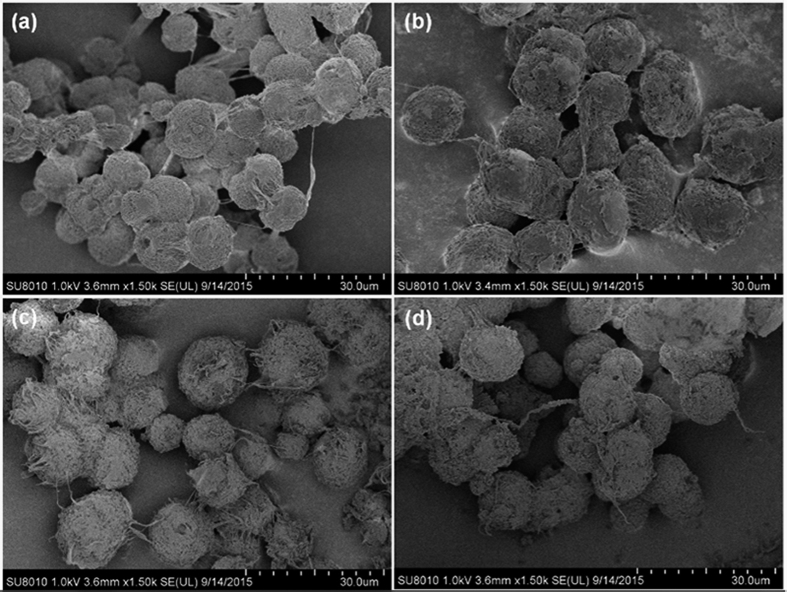
SEM images of (**a**) pure PP, (**b**) PP/10 AMO-LDH, (**c**) PP/10 AMO-LDH‒0.5 OCNT, and (**d**) PP/20 AMO-LDH‒0.5 OCNT.

The morphologies of neat PP, PP/AMO-LDH, and PP/AMO-LDH‒OCNT nanocomposites were further examined using SEM analysis, as shown in Fig. 7. Spherical particles with an average size of about ~10 μm were formed for neat PP and all the PP based nanocomposites. Pure PP showed a very smooth surface. After introducing AMO-LDH or AMO-LDH‒OCNT hybrids, the surface of all the composites became much rougher. The AMO-LDH particles and OCNTs can rarely be observed, which is because of their small size and excellent dispersion within the PP matrix. These data suggest that the AMO-LDH‒OCNT hybrids were well dispersed within the PP matrix through the solvent mixing method.

**Figure 8:**
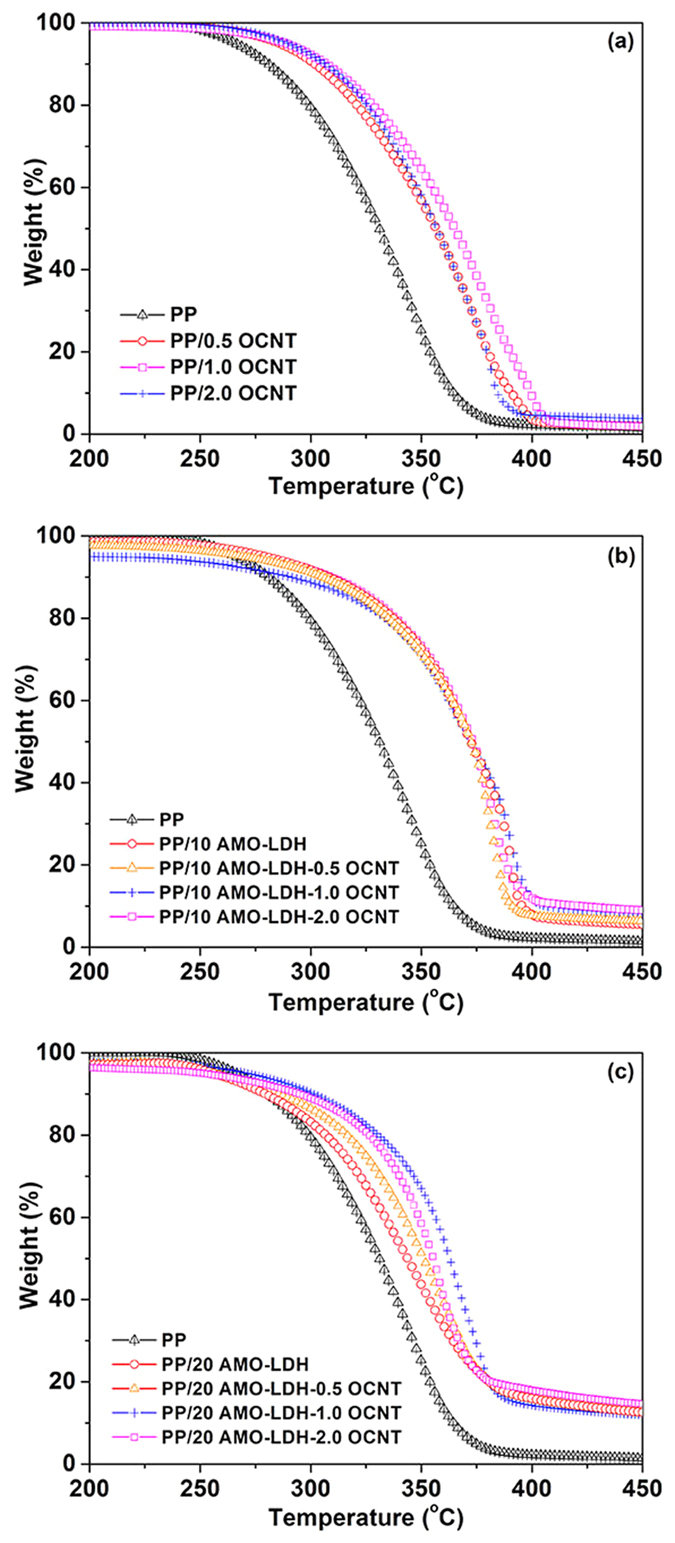
TGA curves of pure PP and (**a**) PP/OCNT, (**b**) PP/AMO-LDH‒OCNT composites with 10 wt% AMO-LDH, and (**c**) PP/AMO-LDH‒OCNT composites with 20 wt% AMO-LDH nanocomposites.

Since both the AMO-LDH nanoplates and OCNTs may affect the thermal stability of PP, all the samples were further investigated using thermogravimetric analysis (TGA). Figure 8 depicts the TGA curves of pure PP, PP/AMO-LDH, and PP/AMO-LDH‒OCNT nanocomposites. The 10% weight loss temperatures (T_0.1_), the 50% weight loss temperature (T_0.5_), and the difference in T_0.1_ and T_0.5_ of all the samples (cf. pure PP) are summarized in Table 3. It is clear that the addition of 10 wt% AMO-LDH to the PP matrix can significantly improve the thermal stability. T_0.1_ and T_0.5_ are increased by 30 and 41 °C, respectively. However, the thermal degradation temperatures (T_0.1_ and T_0.5_) of the sample with 20 wt% AMO-LDH are almost identical to those of pure PP. This is because AMO-LDH is weakly basic and so may catalyze the degradation of PP when its loading is too high^37^. This result is consistent with our previous studies and further demonstrates that high loadings of AMO-LDH nanofillers can result in worsened thermal stability^16^.

Thus, one reason for introducing OCNTs into PP is to reduce the AMO-LDH loading in order to maintain good thermal stability while also improving the flame retardant performance. Table 3 shows that the addition of 0.5, 1, and 2 wt% of OCNT fillers causes the T_0.1_ and T_0.5_ of the nanocomposites to increase. T_0.1_ was increased by 22, 27, and 27 °C, and the T_0.5_ by 25, 34, and 26 °C, respectively (compared to PP alone). A previous study conducted by He *et al*. also obtained similar results, with the addition of 1.0 wt% CNTs into PP increasing T_0.5_ by 34.9 °C^38^. After combining PP with OCNTs together with 10 or 20 wt% of AMO-LDH, T_0.1_ and T_0.5_ were increased by different amounts. For PP/AMO-LDH‒OCNT nanocomposites with 10 wt% AMO-LDH and 0.5, 1, and 2 wt% OCNT, T_0.1_ was increased by 25, 10, and 28 °C while T_0.5_ rose by 41, 41, and 43 °C, respectively. These increases are much higher than observed with PP/AMO-LDH nanocomposites without OCNTs. Similarly, the increase in T_0.1_ of the systems containing 20 wt% AMO-LDH, PP/20 AMO-LDH‒0.5 OCNT, PP/20 AMO-LDH‒1 OCNT, and PP/20 AMO-LDH‒2 OCNT was 5, 21, and 13 °C, while the rise in T_0.5_ was 20, 30, and 24 °C. Figure 8 shows that the degradation in the majority of the samples containing both AMO-LDH and AMO-LDH‒OCNT is slower than neat PP, and the final temperature of decomposition is higher too. Moreover, no decomposition occurs beyond 425 °C for any of the materials, which indicates that a more thermally stable char could be generated at 425 °C after adding OCNTs. This finding could be attributed to the formation of a carbon layer on the surface of the samples^28^. These significant enhancements in decomposition temperatures are attributed to the synergetic effect between LDHs and CNTs.

DTG analyses were carried out to further confirm the decomposition temperatures of pure PP and PP based composites. Figure S1 displays the derivative weight as a function of temperature. The temperature of the maxima in DTG curve of pure PP is around 340 °C, corresponding to its rapid decomposition. It is clear that the temperature of the maxima in DTG rose compared to pure PP after adding AMO-LDH, OCNT or the combination of AMO-LDH and OCNTs. The temperatures of the maxima in DTG lie in the range of 350‒390 °C.

**Figure 9:**
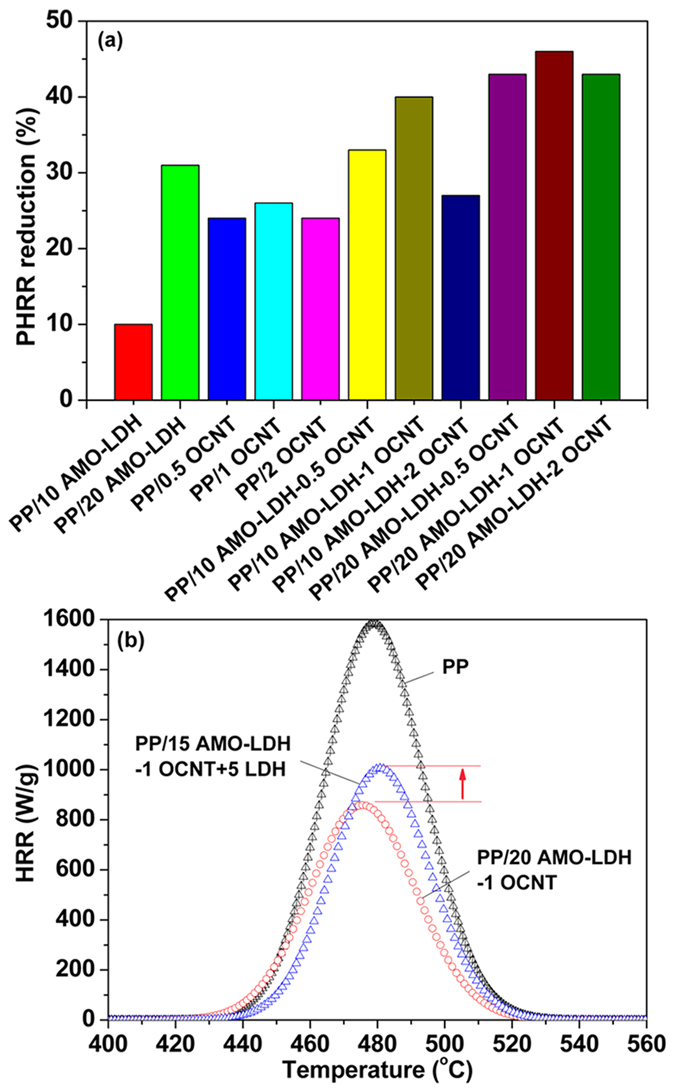
(**a**) PHRR reduction of PP and the various nanocomposites prepared, and (**b**) HRR curves of neat PP, PP/20 AMO-LDH‒1 OCNT, and PP/15 AMO-LDH‒1 OCNT + 5 AMO-LDH nanocomposites.

The flame retardant properties of pure PP and its nanocomposites were evaluated with the microscale combustion calorimeter (MCC‒2) analyzer. The heat release rate (HRR), in particular the peak HRR (PHRR), is the most important parameter in a fire and can be viewed as the “driving force” of combustion. The higher the PHRR is, the more dangerous a material will be if exposed to fire. In order to evaluate the effects of OCNTs on the flame retardation efficiency of AMO-LDH, PP samples containing OCNTs, AMO-LDH, and AMO-LDH‒OCNT hybrids were all investigated. The loadings of AMO-LDH and CNTs and the combustion parameters determined are summarized in Table 4. The PHRR reductions obtained with all samples are presented in Fig. 9. Pure PP is highly flammable, as indicated by a PHRR of 1585 Wg^−1^, total heat release (THR) of 47.6 kJg^‒1^, and heat release capacity (HRC) of 1163 Jg^‒1^K^‒1^. It is clear that the PHRR values of PP/AMO-LDH, PP/OCNT, and PP/AMO-LDH‒OCNT nanocomposites are much lower than that of pure PP. The PHRR value decreased from 1585 to 1196, 1176, and 1197 Wg^−1^ as the loading of OCNTs increased from 0.5, 1, and 2 wt%, corresponding to a PHRR reduction of nearly 24, 26, and 24%, respectively. A similar value was obtained by He *et al*. when introducing 1.0 wt% of CNTs into PP: these authors found that the PHRR decreased from 1513 to 1136 Wg^−1^ (a 24.9% reduction)^38^. These data indicate that OCNTs can improve the flame retardant property of PP. Similarly, the PHRR could also be reduced by the incorporation of AMO-LDH nanoplatelets. With 10 and 20 wt% of AMO-LDH the PHRR declined to 1422 and 1099 Wg^−1^, reductions of 10 and 31%, respectively.

The AMO-LDH‒OCNT hybrids resulted in a much better flame retardant performance: adding even a small amount of OCNTs (0.5 wt%) led to a greater PHRR reduction with the PP/AMO-LDH‒OCNT nanocomposites. For example, with the 10 AMO-LDH‒0.5 OCNT hybrid, the PHRR reduction reached 33%, superior performance than the PP/20 AMO-LDH nanocomposites. Furthermore, with the PP/20 AMO-LDH‒0.5 OCNT hybrid, the PHRR value was decreased by 43%. When the OCNT loading was increased to 1 wt%, the PHRR reductions of the PP/10 AMO-LDH‒1 OCNT and PP/20 AMO-LDH‒1 OCNT nanocomposites were even more profound, at 40 and 46%, respectively. However, for the PP/10 AMO-LDH‒2 OCNT and PP/20 AMO-LDH‒2 OCNT nanocomposites, the PHRR reduction declined to 27 and 43%. Thus, it can be concluded that a balanced amount of OCNT leads to a better flame retardant performance, but excess OCNT hampers the PHRR reduction. In this study, 1 wt% was identified as the optimal OCNT loading.

Another important parameter for fire hazard evaluation is total heat release (THR). Once the ignition takes place, THR steadily increases with burning time and attains a steady state before the flameout occurs. Thus, an efficient flame retardant filler should be able to reduce THR when it is incorporated into a polymer. Table 4 indicates that both PP/AMO-LDH and PP/OCNT nanocomposites result in a lower THR value than neat PP (47.6 kJg^‒1^). For example, the THR value of the PP/AMO-LDH composites decreased to 43.0 and 37.7 kJg^‒1^ with 10 and 20 wt% AMO-LDH loadings, respectively. The THR value for PP/0.5 OCNT, PP/1 OCNT, and PP/2 OCNT decreased to 41.1, 42.8, and 38.4 kJg^‒1^.

After adding the AMO-LDH‒OCNT hybrid filler, the THR was much more significantly reduced. The THR values of 10 and 20 wt% PP/AMO-LDH‒OCNT nanocomposites were only 33‒36 and 28‒31 kJg^‒1^. Considering the fact that AMO-LDH and CNTs cannot release heat, the calculated theoretical THR for the PP/AMO-LDH‒OCNT nanocomposites should be 39.6 and 44.6 kJg^‒1^, respectively: much higher than the experimental values. Most importantly, the THR of the PP/AMO-LDH‒OCNT nanocomposites is much lower than that of the PP/AMO-LDH nanocomposites. These results clearly demonstrate that the AMO-LDH‒OCNT hybrid as flame retardant nanofillers can effectively decrease the THR value of PP.

Similar to the THR, the heat release capacity (HRC) is also significantly reduced after adding AMO-LDH and OCNTs. With 10 and 20 wt% AMO-LDH loading, the HRC declined from 1163 Jg^‒1^K^‒1^ for pure PP to 1071 and 822 Jg^‒1^K^‒1^. These values are lower than the theoretical values of 1135 and 1009 Jg^‒1^K^‒1^ (determined assuming the AMO-LDH does not contribute to HRC). After adding AMO-LDH‒OCNT hybrids to PP, the HRCs of PP/10 AMO-LDH‒0.5 OCNT, PP/10 AMO-LDH‒1 OCNT, and PP/10 AMO-LDH‒2 OCNT nanocomposites were 960, 817, and 1006 Jg^‒1^K^‒1^, respectively. For PP/20 AMO-LDH‒0.5 OCNT, PP/20 AMO-LDH‒1 OCNT, and PP/20 AMO-LDH‒2 OCNT nanocomposites, the HRC value was further decreased to 785, 737, and 775 Jg^‒1^K^‒1^, a significant improvement in performance over the PP/20 AMO-LDH composite (822 Jg^‒1^K^‒1^). These results thus confirm that the AMO-LDH‒OCNT hybrid is very efficient as a flame retardant additive for PP. A possible mechanism underlying the improvement in thermal stability properties and flame retardant performance is that the AMO-LDH‒OCNT hybrid could not only promote char formation, but also mechanically reinforce the char structure as well as increasing the graphitization degree of the char. All these factors make the char act as a physical barrier to retard the transfer of volatile products from the interior of the polymer matrix, and effectively insulate the underlying polymer from the heat source^39^.

We were also interested to learn if the mixing degree between the AMO-LDH and OCNTs could influence the flame retardant properties. To explore this, a 15% AMO LDH/1% OCNT hybrid was synthesized, and this hybrid added together with 5 wt% AMO-LDH into PP. This resulted in an overall loading of AMO-LDH and OCNTs of 20 wt% and 1 wt%. Figure 9(b) shows that the PHRR value of this PP/15 AMO-LDH‒1 OCNT + 5 AMO-LDH nanocomposite was 980 Wg^−1^, reduced by 38% compared to neat PP. However, for the PP/20 AMO-LDH‒1 OCNT nanocomposite, the reduction in PHRR is greater, at 46%. Furthermore, the values of THR and HRC for PP/20 AMO-LDH‒1 OCNT (29.6 KJg^−1^ and 737 Jg^‒1^K^‒1^) are lower than those of PP/15 AMO-LDH‒1 OCNT + 5 AMO-LDH (37.8 KJg^–1^ and 954 Jg^‒1^K^‒1^). Hence, it can be concluded that the degree of mixing between AMO-LDH and OCNT has a significant effect on the flame retardant properties. Better mixing leads to improved flame retardant performance.

Since the addition of large amounts of AMO-LDH may impair the mechanical properties of PP, neat PP, and the PP/10 AMO-LDH, PP/20 AMO-LDH, PP/1 OCNT, PP/10 AMO-LDH-1 OCNT, and PP/20 AMO-LDH‒1 OCNT nanocomposites were investigated using tensile strength measurements. The resultant data are summarized in Table 5. For neat PP, the tensile strength (TS) was 45.7 MPa. However, after adding 10 and 20 wt% of AMO-LDH, the TS decreased to 25.6 and 19.9 MPa, respectively. This is not favorable for subsequent processing step. While when using OCNT as the nanofiller, because its loading was much lower (1 wt%), the TS of PP/1 OCNT remained as high as 37.8 MPa. Surprisingly when using the AMO-LDH‒OCNT hybrid as the filler, the TS values were higher than those of the composites containing only AMO-LDH. For instance, the TS values for PP/10 AMO-LDH‒1 OCNT and PP/20 AMO-LDH‒1 OCNT nanocomposites are 27.5 and 23.8 MPa.

Similarly, the elongation at break (EB) was also significantly decreased by the addition of AMO-LDH, from 51.1% for neat PP to 7.5% and 3.2% for PP/10 AMO-LDH and PP/20 AMO-LDH. However, with the AMO-LDH‒OCNT hybrid, a smaller decrease in the EB was observed, with values of 17.9% and 4.4% for PP/10 AMO-LDH‒1 OCNT and PP/20 AMO-LDH‒1 OCNT. The results therefore demonstrate that OCNTs enabled a greater interfacial stress transfer. The above data also demonstrated that, compared to neat AMO-LDH, the AMO-LDH‒OCNT hybrid is not only better in flame retardancy, but also in maintaining the mechanical properties of PP. For instance, for the PP/10 AMO-LDH‒1 OCNT nanocomposite, its flame retardant performance (40% reduction in PHRR) was better than the PP/20 AMO-LDH nanocomposite (31% reduction in PHRR), and at the same time both its TS (27.5 MPa) and EB (17.9%) were much higher than those of the PP/20 AMO-LDH nanocomposite (TS 19.9 MPa, EB 3.2%).

## Conclusions

A range of aqueous miscible organic [Mg_3_Al(OH)_8_](CO_3_)_0.5_·*y*H_2_O layered double hydroxide‒oxidized carbon nanotube (AMO-LDH-OCNT) hybrids were prepared and evaluated as nanofillers for PP with respect to their thermal stability, flame retardancy, and mechanical properties. All samples were characterized by XRD, SEM, TEM, and zeta potential measurements. SEM and TEM analyses showed that the AMO-LDH particles were attached to the surface of the OCNTs, which can readily disperse in PP to give a homogeneous dispersion. TGA results indicated that the AMO-LDH‒OCNT hybrids could enhance the thermal stability of PP. Particularly for the PP/10 wt% AMO-LDH nanocomposites with 0.5‒2 wt% OCNTs, the temperature at which 50% mass was lost increased by 41‒43 °C, an improved performance over both neat PP and the PP/AMO-LDH nanocomposites. Most importantly, the PP/AMO-LDH‒OCNT nanocomposites possessed of better fire resistance compared with PP/AMO-LDH systems. The peak heat release rate reduction of PP/10 wt% AMO-LDH‒1 wt% OCNT and PP/20 wt% AMO-LDH‒1 wt% OCNT was 40 and 46%, respectively, greater than that of PP/10 wt% AMO-LDH (10%) and PP/20 wt% AMO-LDH (31%) nanocomposites. The extent of mixing between AMO-LDH and OCNT was investigated and it was found that a greater extent of mixing resulted in a better flame retardant performance. The AMO-LDH‒OCNT hybrid additive in PP also led to higher tensile strengths and elongation at break performance for the nanocomposites compared to PP/AMO-LDH nanocomposites.

## Methods

### Preparation of oxidized CNTs

OCNTs were generated by the oxidation of pristine CNTs with nitric acid. In brief, 1 g of CNTs was added to 80 ml of 69% v/v HNO_3_ in water, and the mixture refluxed at 80 °C for 4 h. The obtained OCNTs were washed repeatedly with deionized water until the pH of the filtrate was close to 7, and subsequently dried at 80 °C.

### Synthesis of AMO-LDH–OCNT hybrids

AMO-LDH–OCNTs, based on [Mg_3_Al(OH)_8_](CO_3_)_0.5_·*y*H_2_O (Mg_3_Al–CO_3_ LDH), were synthesized using a similar hydrothermal method as in previous studies^16^,^17^. A metal precursor solution containing 9.60 g Mg(NO_3_)_2_·6H_2_O and 4.70 g Al(NO_3_)_3_·9H_2_O in 50 ml deionized water was added drop-wise into a solution containing 2.65 g Na_2_CO_3_ and the desired amount of OCNTs in 50 ml deionized water, while the pH was kept constant at *ca*. 10 using a NaOH (4 M) solution. The mixture was aged at 65 °C for 30 min, and then the slurry transferred into a 100 ml Teflon–lined autoclave. The autoclave was sealed and a hydrothermal reaction performed for 24 h at 100 °C, followed by product recovery through centrifugation and washing with deionized water until the supernatant pH was close to 7. The AMO-LDH–OCNT hybrids then underwent the aqueous miscible organic solvent treatment (AMOST) by washing the damp solids with acetone several times to remove all the adsorbed water molecules and tune the surface of the AMO-LDH–OCNT hybrids to be hydrophobic. The resultant AMO-LDH–OCNT hybrids were used without drying for the synthesis of PP/AMO-LDH–OCNT nanocomposites via a solvent mixing method.

### Preparation of PP based nanocomposites

PP/AMO-LDH–OCNT nanocomposites were prepared using the solvent mixing method. Firstly PP, AMO-LDH–OCNT, and 100 ml of xylene were charged into a 250 ml round bottom flask. The mixture was refluxed at approximately 140 °C for at least 2 h. After the reflux was complete, the hot xylene solution containing dissolved PP and highly dispersed AMO-LDH–OCNT nanoparticles was poured into 100 ml hexane. The PP/AMO-LDH–OCNT nanocomposites thereby obtained were dried under vacuum. PP/AMO-LDH and PP/OCNT nanocomposites were prepared similarly. Full details of the formulations are listed in Table 1. The composites were prepared by mixing PP with the various AMO-LDH–OCNT hybrids as follows. The 5:1 AMO-LDH-OCNT hybrid was used to make the PP/10 AMO-LDH–2 OCNT composite; 10:1 to make the PP/10 AMO-LDH–1 OCNT and PP/20 AMO-LDH–2 OCNT composites; and, 20:1 to make the PP/10 AMO-LDH–0.5 OCNT and PP/20 AMO-LDH–1 OCNT composite. Finally, the 40:1 AMO-LDH-OCNT material was used to make the PP/20 AMO-LDH–0.5 OCNT composite.

### Characterization

X–ray diffraction (XRD) patterns were recorded on a Shimadzu XRD–7000S instrument in reflection mode with Cu Kα radiation. The accelerating voltage was set at 40 kV with 30 mA current (λ = 1.542 A°). Diffraction patterns were obtained over the range of 2‒70^o^ with a scanning rate of 5° min^–1^. Field emission (FE)–scanning electron microscopy (SEM) analyses were performed on a Hitachi SU–8010 scanning microscope with an accelerating voltage of 5.0 kV. Powder samples were spread on carbon tape adhered to the SEM stage. Before observation, the samples were sputter coated with a thin platinum layer to prevent charging and to improve the image quality. Transmission electron microscope (TEM) analyses were performed on a JEOL 2100 microscope with an accelerating voltage of 400 kV. Samples were dispersed in ethanol with sonication and then casted onto copper TEM grids. Measurements of ζ‒potential were recorded with a Malvern Zetasizer Nano–Z system, after the particles were dispersed in water via ultrasonication for 30 min.

### Thermal stability, flammability properties, and mechanical properties

The thermal stability of all the samples was evaluated using thermogravimetric analysis (TGA Q50 instrument, TA Instruments), with a heating rate of 10 °C min^‒1^ and an air flow rate of 20 ml min^‒1^. DTG is the first derivative curve of TGA, which can obtain by using the software. Further experiments were undertaken from 25 to 600 °C. A microscale combustion calorimeter (MCC-2, Govmark) was used to investigate the combustion behavior of the PP composites. In this system, samples of ca. 5 mg were heated to 700 °C at a heating rate of 1 °C s^‒1^ in a stream of nitrogen flowing at 80 cm^3^ min^‒1^. The volatile, anaerobic thermal degradation products in the nitrogen gas stream were mixed with a 20 cm^3^ min^‒1^ stream of 20% oxygen and 80% nitrogen prior to entering a 900 °C combustion furnace. The parameters measured from this test are the heat release rate (HRR) in Wg^−1^ (calculated from the oxygen depletion measurements), heat release capacity (HRC) in Jg^−1^K^−1^ (obtained by dividing the sum of the peak HRR by the heating rate in °C s^−1^), and the total heat release (THR) in kJg^−1^ (given by integrating the HRR curve). Mechanical properties were quantified using a universal tensile tester (UTM6503, Shenzhen Suns Technology Stock Co. Ltd. China), equipped with a 100 N load cell. At least three measurements were performance at a cross−head speed of 25 mm min^‒1^ and the values are reported as average.

## Additional Information

**How to cite this article**: Gao, Y. *et al*. Layered double hydroxide-oxidized carbon nanotube hybrids as highly efficient flame retardant nanofillers for polypropylene. *Sci. Rep.* **6**, 35502; doi: 10.1038/srep35502 (2016).

## Supplementary Material

Supplementary Information

## Figures and Tables

**Table 1:** The PP based nanocomposites developed.

Sample ID	PP (wt%)	AMO-LDH (wt%)	OCNTs (wt%)	AMO-LDH–OCNT (wt%)
PP	100			
PP/10 AMO-LDH	90	10		
PP/20 AMO-LDH	80	20		
PP/0.5 OCNT	99.5		0.5	
PP/1 OCNT	99		1	
PP/2 OCNT	98		2	
PP/10 AMO-LDH‒0.5 OCNT	89.5			10 AMO-LDH–0.5 OCNT
PP/10 AMO-LDH‒1 OCNT	89			10 AMO-LDH–1 OCNT
PP/10 AMO-LDH‒2 OCNT	88			10 AMO-LDH–2 OCNT
PP/20 AMO-LDH‒0.5 OCNT	79.5			20 AMO-LDH–0.5 OCNT
PP/20 AMO-LDH‒1 OCNT	79			20 AMO-LDH–1 OCNT
PP/20 AMO-LDH‒2 OCNT	78			20 AMO-LDH–2 OCNT
PP/15 AMO-LDH‒1 OCNT + 5 AMO-LDH	79	5		15 AMO-LDH–1 OCNT

**Table 2:** Lattice parameters *a* and *c*, and interlayer distances of the AMO-LDH and AMO-LDH–OCNT hybrids.

Samples	d_003_ (nm)	d_110_ (nm)	*a*^a^ (nm)	*c*^a^ (nm)	ILD^a^ (nm)
AMO-LDH	0.774	0.153	0.306	2.322	0.294
5 AMO-LDH‒1 OCNT	0.778	0.153	0.306	2.334	0.298
10 AMO-LDH‒1 OCNT	0.781	0.153	0.306	2.343	0.301
20 AMO-LDH‒1 OCNT	0.785	0.153	0.306	2.355	0.305
40 AMO-LDH‒1 OCNT	0.778	0.153	0.306	2.334	0.298

^a^Lattice parameters, *c* = 3 d_003_, *a* = 2 d_110_, ILD (interlayer distance) = d_003_–0.48.

**Table 3:** Summary of TGA results obtained with pure PP and its nanocomposites^a^.

Samples	T_0.1_ (^o^C)	ΔT_0.1_ (^o^C)	T_0.5_ (^o^C)	ΔT_0.5_ (^o^C)	Char (wt%)
PP	280	NA	331	NA	0.7
PP/10 AMO-LDH	310	30	372	41	4.7
PP/20 AMO-LDH	280	0	344	13	11.0
PP/0.5 OCNT	302	22	356	25	1.5
PP/1 OCNT	307	27	365	34	1.5
PP/2 OCNT	307	27	357	26	3.3
PP/10 AMO-LDH‒0.5 OCNT	305	25	372	41	6.8
PP/10 AMO-LDH‒1 OCNT	290	10	372	41	7.3
PP/10 AMO-LDH‒2 OCNT	308	28	374	43	7.9
PP/20 AMO-LDH‒0.5 OCNT	285	5	351	20	11.3
PP/20 AMO-LDH‒1 OCNT	301	21	361	30	11.1
PP/20 AMO-LDH‒2 OCNT	293	13	355	24	13.5

^a^T_0.1_ = temperature of 10% mass loss, T_0.5_ = temperature of 50% mass loss; ΔT = difference between the pure polymer and the nanocomposite.

**Table 4:** Summary of combustion data for PP and its nanocomposites^a^.

Samples	PHRR (Wg^–1^)	Reduction (%)	THR (kJg^–1^)	T_max_ (^o^C)	HRC (Jg^–1^K^–1^)
PP	1585	NA	47.6	478.6	1163
PP/10 AMO-LDH	1422	10	43	481.8	1071
PP/20 AMO-LDH	1099	31	37.7	484.5	822
PP/0.5 OCNT	1196	24	41.1	475.8	1080
PP/1 OCNT	1176	26	42.8	475.9	1134
PP/2 OCNT	1197	24	38.4	474.3	1161
PP/10 AMO-LDH‒0.5 OCNT	1061	33	35.5	480.2	960
PP/10 AMO-LDH‒1 OCNT	955	40	32.6	479.9	817
PP/10 AMO-LDH‒2 OCNT	1156	27	34.9	480.9	1006
PP/20 AMO-LDH‒0.5 OCNT	903	43	31.0	477.3	785
PP/20 AMO-LDH‒1 OCNT	856	46	29.6	475.5	737
PP/20 AMO-LDH‒2 OCNT	899	43	27.8	476.4	775
PP/15 AMO-LDH‒1 OCNT + 5 LDH	980	38	37.8	480.8	954

^a^HRC = heat release rate; THR = total heat release; PHRR = peak heat release rate; T_max_ = temperature at maximum pyrolysis rate.

**Table 5:** Mechanical properties of neat PP, PP/OCNT, PP/AMO-LDH, and PP/AMO-LDH‒OCNT nanocomposites.

Samples	Tensile strength (MPa)	Elongation at break (%)
PP	45.7	51.1
PP/10 AMO-LDH	25.6	7.5
PP/20 AMO-LDH	19.9	3.2
PP/1 OCNT	37.8	18.3
PP/10 AMO-LDH‒1 OCNT	27.5	17.9
PP/20 AMO-LDH‒1 OCNT	23.8	4.4
