# Supplementary material for: Layered double hydroxide-oxidized carbon nanotube hybrids as highly efficient flame retardant nanofillers for polypropylene
Source: Sci Rep. 2016 Oct 18;6:35502. doi: 10.1038/srep35502 (PMC5067659; doi:10.1038/srep35502)
Supplement: Supplementary Information [file srep35502-s1.doc]

**Supporting Information**

**Layered double hydroxide-oxidized carbon nanotube hybrids as highly efficient flame retardant nanofillers for polypropylene**

Yanshan Gao^1,2^, Yu Zhang^1^, Gareth R. Williams^2^, Dermot O’Hare^3^, Qiang Wang^1,*^

^1^ College of Environmental Science and Engineering, Beijing Forestry University, 35 Qinghua East Road, Haidian District, Beijing 100083, P. R. China

^2^ UCL School of Pharmacy, University College London, 29-39 Brunswick Square, London WC1N 1AX, United Kingdom

^3^ Chemistry Research Laboratory, Department of Chemistry, University of Oxford, Mansfield Road, Oxford OX1 3TA, United Kingdom

*Corresponding author:

College of Environmental Science and Engineering, Beijing Forestry University, 35 Qinghua East Road, Haidian District, Beijing 100083, P. R. China

Tel.: 86-13699130626

E-mail: [qiangwang@bjfu.edu.cn](mailto:qiangwang@bjfu.edu.cn); [qiang.wang.ox@gmail.com](mailto:qiang.wang.ox@gmail.com)

**Fig. S1** The DTG data of pure (a) PP/OCNT, (b) PP/AMO-LDH‒OCNT composites with 10 wt% AMO-LDH-OCNT, and (c) PP/AMO-LDH‒OCNT composites with 20 wt% AMO-LDH-OCNT.
